# Safeguarding open-weight genomic foundation models through weight locking

**DOI:** 10.64898/2026.07.07.736795

**Published:** 2026-07-10

**Authors:** Aris Karatzikos, Aggeliki Vasilopoulou, Candace SY Chan, Ioannis Mouratidis, Ilias Georgakopoulos-Soares

**Affiliations:** 1 Division of Pharmacology and Toxicology, College of Pharmacy, The University of Texas at Austin, Dell Paediatric Research Institute, Austin, TX, USA; 2 Department of Computer Science, College of Natural Sciences, The University of Texas at Austin, Austin, TX, USA

## Abstract

**Background.:**

Genomic foundation models can dramatically accelerate biological research by learning general-purpose representations of genomic data that transfer across tasks, enabling researchers to predict variant effects, regulatory elements, and molecular function, among others. To safeguard against potential biosecurity threats and malicious misuse of open-weight models, a common strategy involves excluding human-infecting viral genomes from the model’s training corpora. This strategy, however, can be easily circumvented by fine-tuning models on abundantly available viral data. Weight-locking with spectral deformation has been proposed as a potential method to prevent fine-tuning of neural networks, but has not been systematically evaluated in biological AI models.

**Methods.:**

We applied spectral deformation locking to the Evo-1-8k-base genomic foundation model and evaluated a panel of attack configurations spanning naive fine-tuning, low-rank adaptation (LoRA), a simple inserted-layer bypass baseline, and a white-box singular value decomposition (SVD)–chain factorisation at chain lengths *k* ∈ {2,3,5}. Recovered virological capability was quantified on three Human Virome Understanding Evaluation (HVUE) tasks.

**Results.:**

The lock defended against the naive attacker by either standard pipeline. Naive full fine-tuning under the strong lock drove downstream virological capability significantly *below* the pretrained baseline on pathogenicity and host tropism, converting the attack into a capability loss rather than a gain, while naive low-rank adaptation neither moved held-out perplexity (PPL) nor recovered downstream capability above pretrained. Thus, we conclude that by neither route does the naive attacker reach the gain achieved by fine-tuning an unlocked model. Consistent with previous results in non-biological models, an informed attacker who implements the SVD-chain construction does recover capability on pathogenicity prediction, at the cost of increased computational requirements for the fine-tuning process.

**Availability.:**

https://github.com/Georgakopoulos-Soares-lab/glm-locking.

## Introduction

Genomic foundation models have reached a level of biological competence that makes their open-weight release a concrete biosafety concern (Sandbrink, 2023; Pannu et al., 2025; Soice et al., 2023). Evo (Nguyen et al., 2024) is a 7-billion-parameter foundation model pretrained on roughly 300 billion nucleotides of prokaryotic and viral sequences. Although Evo was not trained on human-infecting viruses, it learns to encode virological properties of such viruses such as host tropism, pathogenicity, and transmissibility, directly from raw DNA, as formalised by the Human Virome Understanding Evaluation (HVUE) benchmark (Dutta et al., 2026). These are exactly the properties relevant to pathogen design. The standard biosafety response, filtering pathogen sequences from pretraining data, is insufficient. With 25 000 gradient steps on a modest pathogen corpus, an unlocked copy of Evo measurably improves its held-out viral perplexity from 3.729 to 3.487 and its downstream virological discrimination in roughly one GPU-day of fine-tuning. Because Evo is released with open weights, this attack surface is available to anyone with access to a GPU.

The field currently lacks a principled technical mechanism between full weight release and complete withholding. Capability locking via Spectral Deformation (SpecDef) (Rosati et al., 2025) is a candidate for that middle ground. Before release, selected singular values of target weight matrices are inflated by a factor *α* and paired with a compensation matrix that preserves the forward pass exactly. The resulting ill-conditioned loss landscape obstructs stable fine-tuning: the curvature along the inflated directions scales as 1*/α*^2^, so the stable learning rate is driven sharply down, impeding representational progress within realistic compute budgets. The precise reduction realized in practice is milder than this worst-case scaling, for reasons we quantify in Methods, but the obstruction is sufficient to deter the naive attacker.

The governance question is whether this obstruction is robust against an informed attacker. Rosati et al. (2026) prove a fundamental limit: a white-box attacker can undo convergence-rate control by expanding locked layers into chains of additional linear factors, reducing per-factor spectral norm at linear cost in model size (Theorem 8). Whether this asymptotic defeasibility translates to practical virological capability recovery on a real genomic model has not been empirically tested. The present study closes this gap, and in doing so establishes a second, methodological result: that the standard frozen-feature probing protocol used to benchmark genomic foundation models is confounded by sequence composition and cannot adjudicate this defence, so capability must be measured by fine-tuning.

### Threat model.

We evaluate defence against two attacker tiers. The *naive attacker* possesses Evo’s weights and a small pathogen corpus, runs standard fine-tuning pipelines (full fine-tuning or LoRA (Hu et al., 2022)), and has no specific knowledge of the lock’s mathematical structure. This is the overwhelmingly most common adversary in practice. The *informed attacker* additionally has read Rosati et al. (2026), can implement the Theorem 8 SVD-chain construction at 7-billion-parameter scale, and is willing to pay the resulting compute overhead. We deliberately omit the state-actor-scale adversary with effectively unbounded compute and pathogen-corpus access; against that adversary, no weight-space defence is expected to hold, and other layers of the biosecurity stack must apply.

We applied SpecDef to Evo-1-8k-base at four lock strengths (*α* ∈ {10^4^, 3 × 10^4^, 10^5^, 3 × 10^5^}) and evaluated attack configurations covering five attack classes. Our central finding is that the lock defends against the naive attacker by either standard pipeline: naive full fine-tuning under the strong lock yields a model whose downstream virological capability is significantly below the pretrained baseline on the tasks carrying the strongest signal, and naive low-rank adaptation leaves both perplexity and downstream capability at the pretrained level; by neither route does the naive attacker reach the capability gain achieved by fine-tuning an unlocked model. The informed SVD-chain attacker recovers capability on the task with a clean fine-tuning signal, consistent with Theorem 8, but pays a compute and memory cost to do so. Aggressive fine-tuning diverges immediately, and a simple inserted-layer bypass fails through catastrophic overfitting on the small pathogen corpus. We additionally identify perplexity as an unreliable standalone audit metric and recommend fine-tuning-based capability evaluation as a mandatory governance instrument.

## Methods

### Spectral deformation: construction.

Let $W\in R^{d_{\text{out}}\times d_{\text{in}}}$ have thin SVD *W* = *U*Σ*V*^⊤^. SpecDef constructs a locked weight $\tilde{W}$ and compensation matrix *C* such that $\tilde{W}=U\tilde{\Sigma }V^{⊤}$ and $C=U\left(\Sigma {\tilde{\Sigma }}^{-1}\right)U^{⊤}$, where ${\tilde{\sigma }}_{i}=\alpha \sigma_{i}$ for the top *k*_lock_ singular values and ${\tilde{\sigma }}_{i}=\sigma_{i}$ otherwise. The forward pass $C\tilde{W}x=Wx$ is preserved to floating-point precision. By the Hessian spectral lower bound of Rosati et al. (2026) (Theorem 3), inflation raises $\sigma_{\text{max}}(\tilde{W})\approx \alpha \sigma_{1}$, scaling the Hessian curvature along the dominant singular direction to $𝒪\left(\alpha^{2}\right)$ and forcing stable optimisation to use a learning rate *η* ≲ 1*/*(*ασ*_1_)^2^. We present the *α*^2^ relation as the theoretical curvature scaling; the *realised* stable learning rate is substantially milder than this worst-case bound because only the top *k*_lock_ = 25 of 4096 singular directions are inflated, because the 8-bit AdamW optimiser normalises updates per coordinate, and because gradients are clipped to unit norm. Empirically, at *α* = 3 × 10^4^ (the primary lock used throughout this study) the realised stable rate is *≈* 10^*−*5^ and naive fine-tuning at 10^*−*4^ diverges. Theorem 8 of Rosati et al. (2026) implies a constructive bypass: a locked layer can be expanded into a chain of *k* jointly-trainable linear factors, reducing per-factor spectral norm from $\sigma_{\text{max}}(\tilde{W})$ to $\sigma_{\text{max}}(\tilde{W}{)}^{1/k}$ at linear cost in model size. We implement this as an SVD-chain factorisation, initialising $\tilde{W}\approx L_{k}\cdots L_{1}$ from matrix-root approximations of the locked weight. For *k*=3 at *α* = 3 × 10^4^, each factor has *σ*_max_ *≈* 47.5 rather than 10^5^, restoring stable gradient flow at *η* = 10^*−*5^. Because the per-factor norm scales as the *k*-th root of *α*, raising the lock strength imposes only a sub-linear increase in the chain length the informed attacker must use; consistent with this, the parameter overhead reported by Rosati et al. (2026) grows roughly logarithmically in *α*. Lock strength is therefore an effective lever against the naive attacker but a weak one against the informed SVD-chain attacker.

### Model and locking.

Evo-1-8k-base (Nguyen et al., 2024) has 7 B parameters across 32 blocks (29 Hyena convolutional operators and 3 multi-head attention layers), hidden dimension 4096, and a single-nucleotide tokenizer. SpecDef was applied to the 32 write-side output projections (the per-block out_filter_dense and attention out_proj matrices, each 4096 × 4096), inflating the top *k*_lock_ = 25 singular values by *α* and fusing *C* into the release checkpoint. SpecDef leaves the number and choice of locked layers as a free parameter of the construction (Rosati et al., 2026); their own experiments range from a single layer on Llama-3.1-8B to full reparameterisation on SmolLM2-360M, with random layer selection as the default. We instead selected the write-side output projections systematically, on the hypothesis that obstructing the block-output composition point is disproportionately effective per locked parameter. These 32 projections comprise approximately 537 M parameters. Four locked checkpoints were produced with *α* ∈ {10^4^, 3 × 10^4^, 10^5^, 3 × 10^5^}, with *α* = 3 × 10^4^ used as the primary lock strength. In all four, the maximum perplexity drift before any fine-tuning is below 3 × 10^*−*4^, confirming exact forward-pass preservation.

### Attack corpus and locking fine-tuning.

We assembled 910 NCBI Virus assemblies of human-pathogenic viruses (including Influenza A, SARS-CoV-2, Ebola, HIV, Dengue, West Nile, and others), split by accession into 544 training and 366 held-out genomes with no taxonomic overlap. All locking runs used 8-bit AdamW (Dettmers et al., 2022), gradient clipping to norm 1.0, fp32 precision, 1024-nt windows with 512-nt stride, batch 1, gradient-accumulation steps 4, on a single NVIDIA A100 80 GB, for 25 000 steps of next-token prediction on the viral corpus. An unlocked control (*η* = 10^*−*5^) establishes the attacker’s performance ceiling at PPL 3.487.

### Capability evaluation by fine-tuning.

Virological capability was measured on three binary HVUE tasks (Dutta et al., 2026): Host Tropism, Pathogenicity, and Transmissibility. The standard benchmark protocol for genomic foundation models extracts frozen final-layer activations, mean-pools them, and trains a linear probe (Marin et al., 2024). We found this protocol to be confounded by sequence composition: a 4-mer-frequency logistic-regression baseline (trained on 3000 sequences and evaluated on 2000) attains area under the receiver operating characteristic curve (AUROC) 0.903*/*0.846*/*0.914 across the three tasks, *exceeding* the probed Evo representations. The probing protocol also produced an implausible signal reversal in which a naively attacked checkpoint scored above the unlocked ceiling (Fig. S2). We therefore evaluate capability by fine-tuning each attacked checkpoint with low-rank adapters (LoRA (Hu et al., 2022), rank 16, *α*_LoRA_ = 32) on each HVUE task for up to 5000 steps with a binary classification head. This setup reflects what a downstream attacker would actually do and recovers genuine model-borne signal: LoRA fine-tuning beats the *k*-mer baseline on every task, improving Matthews correlation coefficient (MCC) by 0.08 to 0.33 (Table 1). Each configuration was run with three random seeds. Statistical significance was assessed by paired bootstrap (*n* = 10000), resampling the held-out evaluation set with a common index set for both checkpoints, against the pretrained baseline; we report AUROC and MCC at the Youden-optimal threshold.

### Attack configurations.

The attacks span five classes, each run for 25 000 locking steps. *Naive fine-tuning* is evaluated at three learning rates: the lowest rate tested, at which training remained stable without divergence *η* = 10^*−*6^, the standard fine-tuning rate a typical practitioner would use without the lock *η* = 10^*−*5^, and an aggressive rate *η* = 10^*−*4^ at the divergence threshold. The principal naively-attacked checkpoint (denoted *M*) is produced under the strong lock *α* = 3 × 10^5^ at the standard fine-tuning rate *η* = 10^*−*5^, i.e. the rate a practitioner unaware of the lock would use by default. *LoRA-locking* attaches rank-16 adapters to the backbone during the 25 000-step viral fine-tuning (distinct from the LoRA used for downstream capability evaluation). *Inserted-layer bypass baseline* inserts an identity-initialised 4096 × 4096 trainable matrix *B* after each frozen fused $C\tilde{W}$, adding 537 M parameters; gradients flow to *B* independently of the lock’s spectral structure, but the locked weight is not factorised and its spectral norm is not reduced. *SVD-chain factorisation* (Theorem 8, *k* ∈ {2,3,5}) replaces each $\tilde{W}$ with *k* jointly-trainable factor matrices initialised from the *k*-th matrix root of $\tilde{W}$, reducing per-factor spectral norm to $\sigma_{\text{max}}(\tilde{W}{)}^{1/k}$ and restoring stable gradient flow at *η* = 10^*−*5^. The compensation matrix *C* is released as a fixed, non-trainable component of the checkpoint and is not updated during the attack, so only the factor matrices *L*_1_*,…,L*_*k*_ are trained; this reflects a standard fine-tuning pipeline in which the attacker trains the model weights but not the fixed compensation structure. An informed attacker could in principle co-adapt *C* as well, but because the factor matrices already span the weight space of each locked layer, training *C* is not expected to expand the set of reachable configurations; we do not evaluate this variant and note it in the Limitations. *Lock-strength robustness conditions* re-run naive fine-tuning at *η* ∈ {10^*−*5^, 10^*−*6^} under the higher locks to assess how the operating point shifts with *α*. The three rates characterise qualitatively distinct optimisation regimes rather than exhaustively sampling a continuous axis. Across the rates tested, *η* = 10^*−*5^ under the strong lock produces the strongest downstream performance for the naive attacker and constitutes the principal naively-attacked checkpoint *M*; even so, *M* remains significantly below the pretrained baseline on Pathogenicity and Host Tropism (Table 1), and well below the unlocked fine-tuning ceiling. Rates below *η* = 10^*−*5^ move the locked weights less and yield results closer to or at the pretrained level without recovering capability, as the LoRA-locking condition illustrates. We did not perform a finer grid search between *η* = 10^*−*6^ and *η* = 10^*−*5^; however, since the stronger of the two tested rates already fails to recover capability, intermediate rates would be expected to produce results on a continuum between two unfavourable outcomes for the attacker, and *M* represents the most favourable naive operating point within the tested range. The full attack taxonomy with held-out perplexities is reported in Supplementary Table S1; per-step wall-clock cost, parameter counts, and memory footprints are in Supplementary Table S2. The full attack taxonomy with held-out perplexities is reported in Supplementary Table S1; per-step wall-clock cost, parameter counts, and memory footprints are in Supplementary Table S2.

### Notation and abbreviations.

We write $W\in R^{d_{\text{out}}\times d_{\text{in}}}$ for a weight matrix with output and input dimensions *d*_out_*, d*_in_, and *W* = *U*Σ*V*^⊤^ for its singular value decomposition (SVD), where *U* and *V* are orthogonal and Σ = diag(*σ*_1_ ≥ *σ*_2_ ≥⋯) holds the singular values; *σ*_1_ *≡ σ*_max_ denotes the largest. The lock inflates the top *k*_lock_ singular values by the *inflation factor α*, producing the locked weight $\tilde{W}$ (with inflated spectrum $\tilde{\Sigma }$) and a compensation matrix *C*. The SVD-chain attack factorises $\tilde{W}$ into *k* linear factors *L*_*k*_ ⋯*L*_1_, where the *chain length k* ∈ {2,3,5} is distinct from *k*_lock_ (the number of inflated directions, fixed at 25) and from the 4-mer (*k*-mer with *k*=4) composition baseline. We write *η* for the learning rate. For low-rank adaptation (LoRA), the adapter scaling parameter *α*_LoRA_ is unrelated to the lock inflation factor *α*. Capability is reported as the AUROC and the Matthews correlation coefficient (MCC), evaluated at the Youden-optimal threshold; held-out viral perplexity is abbreviated PPL. Statistical comparisons use a paired bootstrap with *n* resamples.

## Results

### Forward-pass preservation.

After SpecDef at all four lock strengths, held-out viral PPL is 3.729, identical to the pretrained baseline within |Δ| < 0.001. The compensation matrix exactly cancels the spectral inflation at inference: per-token held-out negative log-likelihood of each locked model lies on the identity against the pretrained model at every lock strength (Pearson *r* ≥ 0.9999; Supplementary Fig. S1), so on any zero-shot task the locked model is indistinguishable from the pretrained one. The lock therefore acts entirely on the optimisation problem, not on the model’s deployed behaviour, and any defensive or adversarial effect manifests only after a fine-tuning attempt.

### Frozen-feature probing is confounded by composition.

Before evaluating the defence, we establish that the standard probing protocol cannot measure it (Supplementary Fig. S2). A 4-merfrequency baseline attains AUROC 0.903*/*0.846*/*0.914 and MCC 0.662*/*0.549*/*0.733 on Host Tropism, Pathogenicity, and Transmissibility, exceeding the probed Evo representations on all three tasks: probed pretrained Evo reaches only AUROC 0.860*/*0.815*/*0.852 and MCC 0.572*/*0.455*/*0.538. A benchmark on which a context-free *k*-mer count outperforms a 7-billion-parameter foundation model is measuring nucleotide composition, not learned virological structure. Probing additionally produced a qualitatively impossible result: a naively-attacked locked checkpoint scored *above* the unlocked fine-tuning reference, a signal reversal inconsistent with any coherent notion of capability recovery. Fine-tuning evaluation removes both pathologies. Under LoRA fine-tuning, Evo beats the *k*-mer baseline on every task (Table 1), by up to +0.134 AUROC and +0.325 MCC on Pathogenicity, and the rankings become monotone in attack strength. All capability results below therefore use LoRA fine-tuning; the probing numbers are retained in the Supplement only to document the confound.

### The lock defends against the naive attacker.

**Figure 1:**
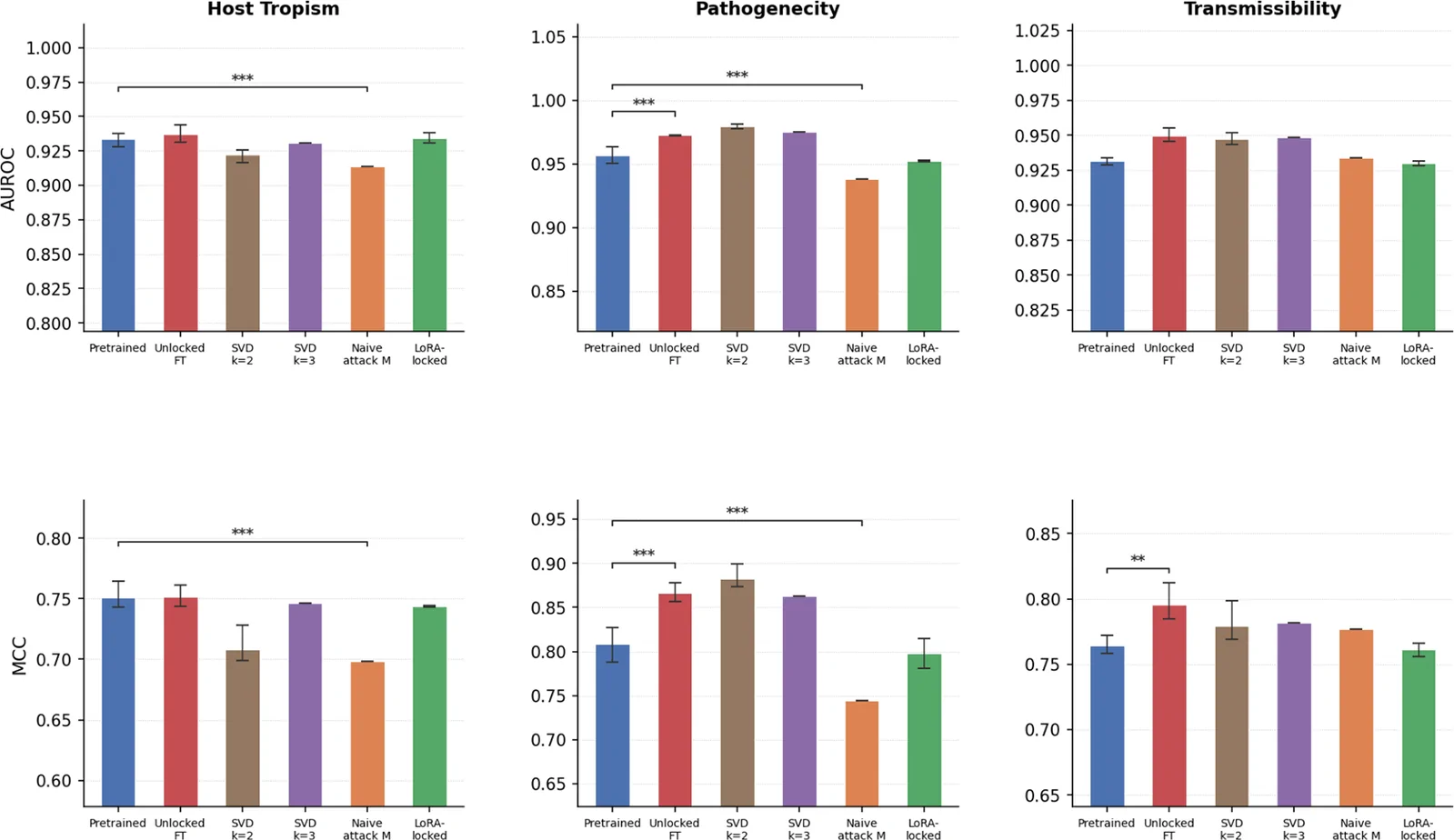
Downstream virological capability under LoRA fine-tuning. **(a)** Mean HVUE AUROC and **(b)** MCC across three seeds for the pretrained reference, the unlocked fine-tuned checkpoint, the informed SVD-chain attacks (*k*=2, *k*=3), and the two naive-attacker pipelines, full fine-tuning under the strong lock (*M*) and LoRA-locking; error bars span the seed range. On Pathogenicity, the unlocked and SVD-chain checkpoints are significantly above pretrained while *M* falls significantly below and LoRA-locking sits at the pretrained level; Host Tropism is near-saturated and Transmissibility separates on neither axis robustly.

Our central result is read directly from Table 1 and Fig. 1, and holds for both pipelines a naive attacker would use. The naively-attacked checkpoint *M* — produced by 25 000 steps of standard full fine-tuning under the strong lock at the default rate *η* = 10^*−*5^ — is significantly *below* the pretrained baseline on Pathogenicity and Host Tropism across both metrics: on Pathogenicity, the task carrying the clearest signal, *M* falls to AUROC 0.938 (*−*0.019, *p* < 10^*−*3^) and MCC 0.744 (*−*0.065, *p* < 10^*−*3^), and on Host Tropism to AUROC 0.914 (*−*0.019, *p* < 10^*−*3^); on Transmissibility it is statistically indistinguishable from pretrained (+0.002, n.s.). The second naive pipeline, low-rank adaptation of the backbone during locking (LoRA-locking), neither moves held-out perplexity (3.729, identical to pretrained; Table S1) nor recovers downstream capability above pretrained on any task (Table 1): the locked spectral geometry constrains what a rank-16 additive update can contribute, so the attack is inert rather than damaging. By neither route, therefore, does the naive attacker reach the capability gain that fine-tuning an unlocked model achieves. For full fine-tuning the lock does more than deny that gain — it converts the attempt into a capability loss, yielding a model worse at the dangerous tasks than the unmodified one. Mechanistically, at a learning rate at or below the realised stable threshold the gradient steps inject noise into the locked projections faster than they drive the representational reorganisation virological tasks require; lowering the rate further mainly reduces movement, so no rate available to the naive attacker escapes this regime. The contrast with the unlocked attacker is the defence: identical full fine-tuning of an unlocked model raises Pathogenicity capability significantly above pretrained (AUROC 0.973, +0.016, *p* < 10^*−*3^; MCC 0.867, +0.058, *p* < 10^*−*3^), exactly the gain the lock denies. That the locked checkpoint without any attack (Locked, no FT) sits essentially at pretrained (Δ within seed noise) confirms the deficit in *M* is produced by the attack interacting with the lock, not by the lock degrading the representation on its own.

### The informed attacker recovers capability, at a cost.

**Figure 3:**
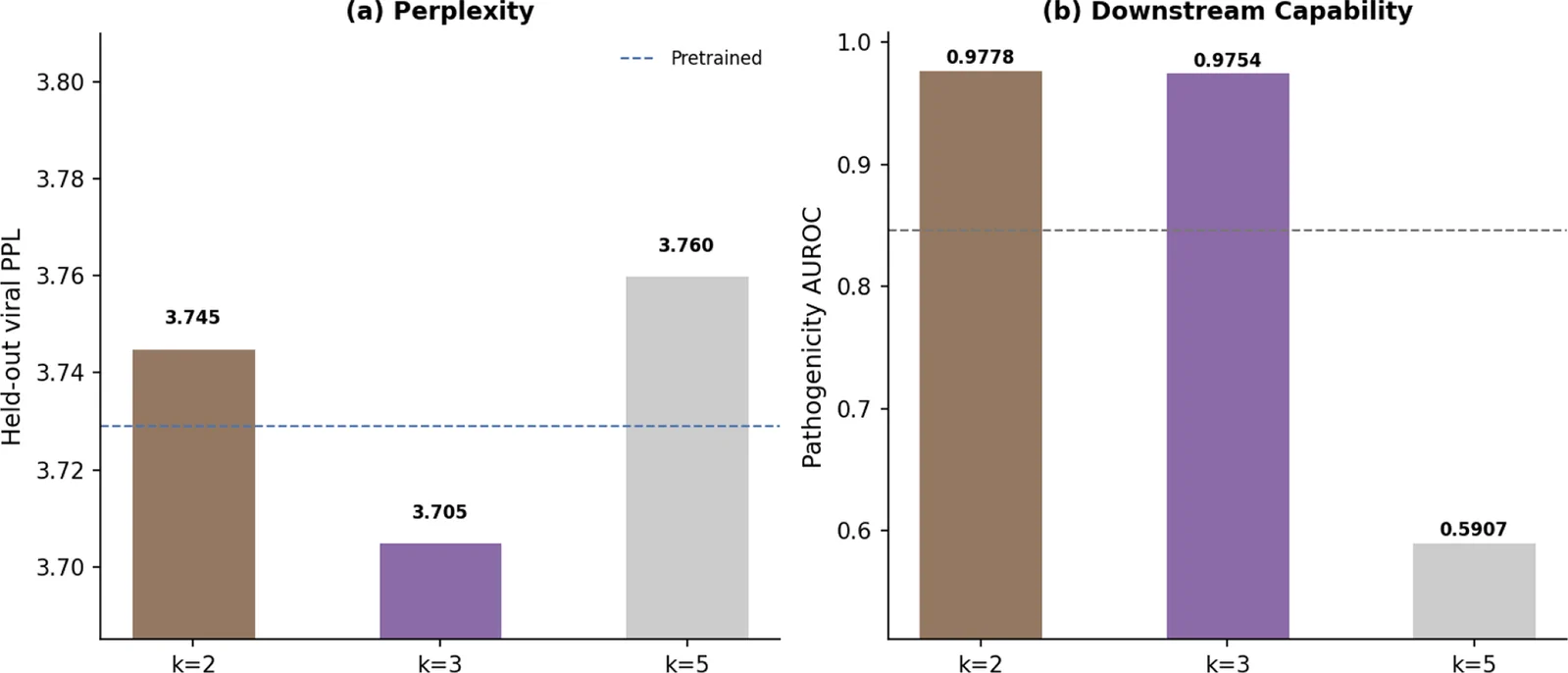
SVD-chain *k*-ablation under the primary lock (*α* = 3 × 10^4^). Downstream Pathogenicity AUROC **(a)** and held-out viral perplexity **(b)** for chain lengths *k*=2, *k*=3, and *k*=5. All are genuine Theorem 8 constructions with per-factor spectral norm reduced to $\sigma_{\text{max}}(\tilde{W}{)}^{1/k}$. Chain length *k*=2 — the minimal non-trivial expansion — attains the highest downstream capability and recovers perplexity toward pretrained; *k*=5 offers no improvement and degrades, indicating the construction saturates by *k*=3. Increasing *k* monotonically increases the attacker’s parameter and wall-clock cost (Supplementary Table S2).

We evaluate SVD-chain factorisation as an informed attack, requiring implementation of the Theorem 8 layer-expansion construction at 7-billion-parameter scale Rosati et al. (2026). On Pathogenicity it recovers capability fully: *k*=2 reaches AUROC 0.980 and MCC 0.882, statistically at or above the unlocked fine-tuned checkpoint (0.973/0.867) and significantly above pretrained (*p* < 10^*−*3^); *k*=3 reaches AUROC 0.975. This is the expected outcome: Theorem 8 guarantees that a white-box attacker can restore trainability by distributing the inflated spectral norm across *k* factors, and our results confirm that the guarantee is realised in practice on a genomic model. Relative to unlocked fine-tuning, the SVD-chain attack pays 1.60 × (*k*=2), 1.91 × (*k*=3), and 2.51 × (*k*=5) the per-step wall-clock time, and adds 1.07–2.68 billion trainable parameters, raising the GPU memory footprint by +8.1 to +23.6GB above the unlocked baseline under fp32 weights with 8-bit AdamW (Supplementary Table S2); at *k*=5 the extra footprint alone consumes nearly a third of an A100 80GB. Chain length *k*=2 is the minimal non-trivial expansion and is empirically the strongest on Pathogenicity (Fig. 3); *k*=5 offers no further gain in this implementation.

### Aggressive fine-tuning and the inserted-layer bypass fail.

Two further attacks fail outright. Aggressive fine-tuning at *η* = 10^*−*4^ (100 × the realised stable locked rate) causes permanent gradient explosion from step 100: gradient norms reach inf and never recover, confirming the curvature barrier is a hard constraint at this rate rather than a soft penalty that more compute could overcome. The inserted-layer bypass, which routes gradients around the locked weight without reducing its spectral norm, produces results *worse* than the naively-locked baseline on both the perplexity and capability axes: 537 M inserted parameters trained on *≈*550 000 tokens, roughly 1000 parameters per training token, memorise the attack corpus without generalising. Removing the gradient-flow obstruction is therefore insufficient; the attacker must reduce the spectral norm itself, which is precisely what the SVD-chain construction does and the bypass does not.

### Perplexity is not an informative standalone audit metric.

Held-out perplexity does not reliably track downstream virological capability across attacks. The naive strong-lock full-FT attack *M* is worse on both axes (PPL 3.810 and capability below pretrained) and the unlocked model improves on both, but the broader pattern is not a stable correlation. Two discordances make this concrete. First, the SVD-chain attacks recover downstream Pathogenicity capability to the unlocked level while their held-out perplexity recovers only to roughly the pretrained regime (3.705–3.760) rather than the unlocked target (3.487): capability is recovered where perplexity is not. Second, naive LoRA-locking leaves perplexity exactly at the pretrained value (3.729) yet likewise produces no downstream gain: an auditor seeing an unchanged perplexity could not distinguish this inert attack from no attack at all. Perplexity is therefore sensitive to next-token adaptation on the viral corpus but is not a reliable proxy for the model-borne representations that downstream discrimination requires. The residual perplexity gap observed for the SVD-chain attack should accordingly not be interpreted as a robust defensive barrier, especially as no hyperparameter optimisation of the SVD attack was performed and a tuned attacker might close it. Perplexity remains useful as a coarse check of whether an attack altered the language-modelling objective, but it is not sufficient for governance conclusions. Fine-tuning-based capability evaluation on benchmarks such as HVUE (Dutta et al., 2026) is a mandatory complement to perplexity for any release-time audit of a locked genomic model.

## Discussion

The empirical result of this study is a clean separation by attacker tier. Against a naive attacker, who downloads the weights and runs a standard fine-tuning pipeline, spectral-deformation locking is an effective defence. Under the strong lock, 25 000 steps of naive full fine-tuning yield a model whose downstream virological capability is significantly below the pretrained baseline on Pathogenicity and Host Tropism, which are the tasks where the signal is strongest, converting the attack from a capability gain into a capability loss, and naive low-rank adaptation leaves both perplexity and downstream capability at the pretrained level. Against the informed attacker who implements the Theorem 8 SVD-chain construction, the lock does not prevent recovery, on the task with a clean fine-tuning signal the informed attack reaches the unlocked level, exactly as the asymptotic theory predicts, but it imposes a quantifiable cost: 1.6 to 2.5 × the per-step wall-clock time and up to +23.6 GB of GPU memory. The governance value of the lock is thus twofold: it defends outright against the common attacker, and it raises the compute and skill floor for the sophisticated one.

### Relation to the asymptotic limit.

Our findings are consistent with, and a practical instantiation of, the limits proved by Rosati et al. (2026): convergence-rate control as a class is asymptotically defeasible by a white-box adversary at linear cost in model size. The SVD-chain recovery on downstream tasks confirms this result on a genomic model as well. The relevant question for biosecurity governance is not whether locking is asymptotically defeasible but whether it provides meaningful deterrence at the data and compute scales of a realistic pathogen-design attacker. Our results indicate that it does against the naive attacker and that it imposes some cost on the informed one, while leaving inference capabilities unchanged.

### Compute and memory cost as defensive quantities.

For the informed attacker the lock converts an absolute barrier into a cost barrier on three axes simultaneously: it caps the capability reachable at a given chain length, raises wall-clock time per step, and inflates the hardware footprint required to mount the attack (Supplementary Table S2).

### A methodological caution for genomic model auditing.

A secondary but practically important contribution is negative: the frozen-feature probing protocol routinely used to benchmark genomic foundation models is confounded by sequence composition. On all three HVUE tasks a context-free 4-mer baseline outperformed probed Evo representations, and probing produced an impossible signal reversal in which a naively-attacked checkpoint scored above the unlocked reference. Any safety audit that relied on probing would have drawn the wrong conclusion about whether capability had been recovered. Fine-tuning-based evaluation removes the confounding factor; Evo beats the *k*-mer baseline by up to 0.33 MCC once adapted, and is the protocol we recommend for capability auditing of locked genomic models. Perplexity is likewise insufficient on its own: a working lock suppresses capability while barely moving perplexity.

### Toward targeted unlearning.

The motivation for complementary defences is not only that locking fails to prevent recovery by the informed SVD-chain attacker, but also that it is indiscriminate: the ill-conditioned loss landscape blocks all fine-tuning on the locked weights equally, preventing legitimate adaptation for safe applications such as clinical variant interpretation, gene-function annotation, or regulatory-element prediction. Targeted machine unlearning is a technique that identifies the layers where virological representations are most concentrated via layer-wise HVUE probing and causal activation patching (Meng et al., 2022), then applies gradient difference (Liu et al., 2022) or representation misdirection (Li et al., 2024) restricted to those layers and would remove the dangerous capability from the weights while preserving unrelated adaptability. It would therefore target the biosecurity-relevant capability directly, rather than obstructing fine-tuning in general; if the information itself were removed, an SVD-chain reparametrisation would have to relearn it rather than merely restore trainability. The unlocked attacker result reported here, recovering significant Pathogenicity capability from 544 training genomes in 25,000 steps, establishes a concrete relearning-attack baseline against which any future unlearning evaluation on Evo-class models should be validated.

### Limitations.

The 25 000-step locking runs that produce each attacked checkpoint are single-seed because each requires 36–57 GPU-hours and the full suite consumed several hundred GPU-hours. This evaluation covers a single model (Evo-1-8k-base), a single locking method (SpecDef), and a human-pathogen corpus of 910 assemblies; generalisation to pure-transformer genomic models (Dalla-Torre et al., 2025; Zhou et al., 2024), to other locking approaches, and to larger attack corpora are open questions. We do not evaluate an informed attacker who additionally co-adapts the compensation matrix *C* during the SVD-chain attack; because the factor matrices already span the weight space of each locked layer, training *C* is not expected to expand the set of reachable configurations, but this remains untested.

## Supplementary Material

1

## Figures and Tables

**Figure 2:**
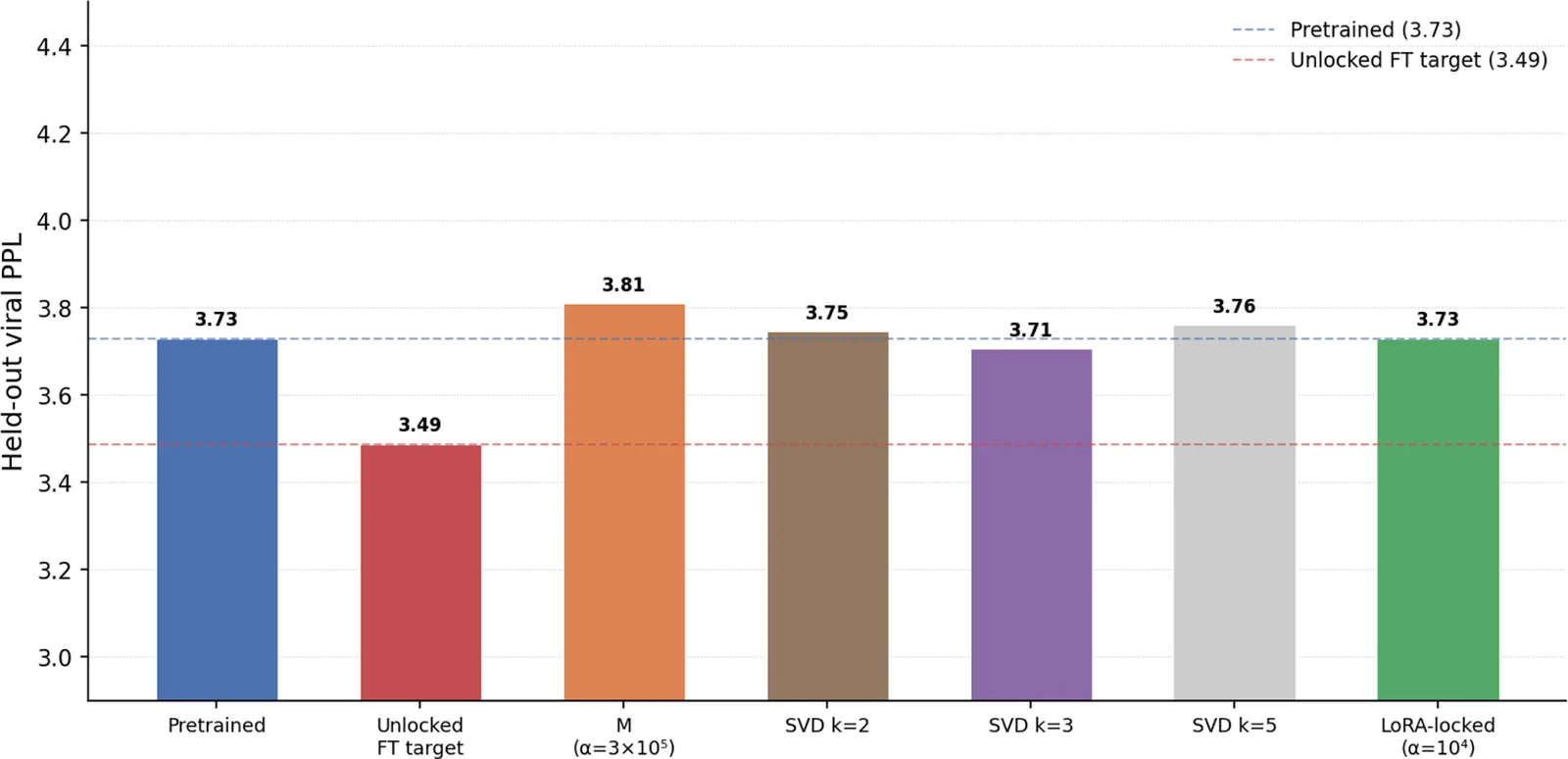
Held-out viral perplexity across representative conditions. Dashed lines mark the pretrained baseline (PPL 3.729) and the unlocked fine-tuning target (PPL 3.487). Unlocked fine-tuning improves held-out viral perplexity, whereas the naive strong-lock full-FT attack *M* degrades it. The SVD-chain attacks recover perplexity only to approximately the pretrained regime rather than to the unlocked target, and naive LoRA-locking leaves perplexity unchanged at the pretrained value. These values are reported as language-modelling diagnostics only; downstream HVUE fine-tuning, not perplexity alone, is used to assess recovered virological capability.

**Table 1:** Downstream HVUE capability under LoRA fine-tuning (full configuration). Mean AUROC and MCC across three seeds. The *k*-mer row is a 4-mer-frequency logistic-regression baseline (AUROC and MCC from the same fit; train 3000, evaluate 2000). Significance by paired bootstrap (*n* = 10000) against the pretrained reference. The two naive-attacker pipelines are grouped: naive full fine-tuning under the strong lock (*M*, *α* = 3 × 10^5^, *η* = 10^*−*5^) is significantly below pretrained on Pathogenicity and Host Tropism, while naive LoRA-locking (*α* = 10^4^) sits at the pretrained level on all tasks. The unlocked and SVD-chain checkpoints are significantly above pretrained on Pathogenicity.

	Host Tropism		Pathogenicity		Transmissibility	
Checkpoint	AUROC	MCC	AUROC	MCC	AUROC	MCC
*k*-mer baseline	0.903	0.662	0.846	0.549	0.914	0.733
*Reference conditions*						
Pretrained	0.933	0.751	0.957	0.809	0.932	0.765
Locked, no FT	0.938	0.744	0.954	0.792	0.943	0.774
Unlocked FT	0.937	0.752	0.973***	0.867***	0.949	0.796**
*Naive attacker (no knowledge of the lock)*						
Full FT, strong lock (*M*)	0.914***	0.698***	0.938***	0.744***	0.934	0.777
LoRA-locking	0.935	0.744	0.952	0.798	0.930	0.761
*Informed attacker (SVD-chain, Theorem 8)*						
SVD-chain *k*=2	0.923	0.708	0.980***	0.882***	0.948	0.779
SVD-chain *k*=3	0.931	0.746	0.975***	0.863***	0.949	0.782

* *p*<0.05;

** *p*<0.01;

*** *p*<10^−3^; unmarked differences are not significant (*p* ≥ 0.05).

## Data Availability

The HVUE benchmark is publicly available (Dutta et al., 2026). The pathogen attack corpus accession lists, SpecDef locking scripts, fine-tuning configurations and HVUE evaluation pipeline are available at https://github.com/Georgakopoulos-Soares-lab/glm-locking. Evo-1-8k-base weights are from the original authors; we redistribute no model weights.
