# Supplementary material for: Safeguarding open-weight genomic foundation models through weight locking
Source: bioRxiv. 2026 Jul 10:2026.07.07.736795. Preprint. [Version 1] doi: 10.64898/2026.07.07.736795 (PMC13370401; doi:10.64898/2026.07.07.736795)
Supplement: Supplementary file 1 [file NIHPP2026.07.07.736795V1-supplement-1.pdf]

bioRxiv preprint doi: https://doi.org/10.64898/2026.07.07.736795; this version posted July 10, 2026. The copyright holder for this preprint (which was not certified by peer review) is the author/funder, who has granted bioRxiv a license to display the preprint in perpetuity. It is made available under a CC-BY 4.0 International license.

Supplementary Material

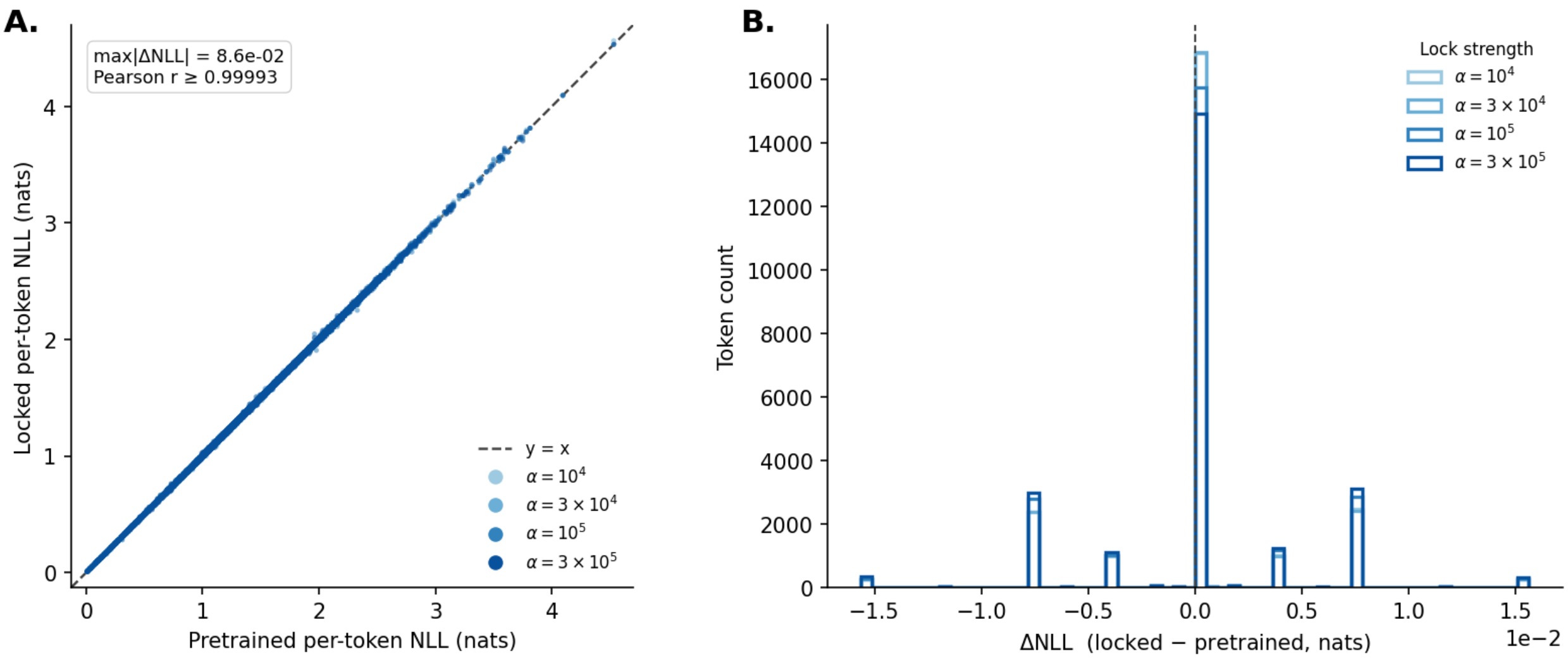

Figure S1: SpecDef preserves the forward pass at all four lock strengths. Per-token held-out negative log-likelihood (NLL) of each locked model against the pretrained model on 24,552 held-out viral tokens. Each checkpoint's compensation is fused in float64 (as in the release checkpoint; per-element reconstruction error $< 2 \times 10^{-9}$) and evaluated in bfloat16. **(a)** All four lock strengths ($\alpha \in \{10^4, 3{\times}10^4, 10^5, 3{\times}10^5\}$) lie on the identity line (Pearson $r \geq 0.9999$; max $|\Delta\text{NLL}| = 0.09$ nats). **(b)** The deviation from pretrained is centred at zero with no dependence on α;

Probing versus fine-tuning evaluation. Supplementary Table S1 reports held-out viral perplexity for every locking condition; PPL is unaffected by the composition confound and these values are the canonical perplexity reference for the study. The composition confound in frozen-feature probing is documented in Supplementary Fig. S2, which contrasts probing and LoRA-fine-tuning capability against the 4-mer baseline: under probing the k-mer baseline exceeds Evo and a naively-attacked checkpoint scores above the unlocked reference, whereas under fine-tuning Evo clears the baseline and the ranking is monotone in attack strength.

bioRxiv preprint doi: https://doi.org/10.64898/2026.07.07.736795; this version posted July 10, 2026. The copyright holder for this preprint (which was not certified by peer review) is the author/funder, who has granted bioRxiv a license to display the preprint in perpetuity. It is made available under a CC-BY 4.0 International license.

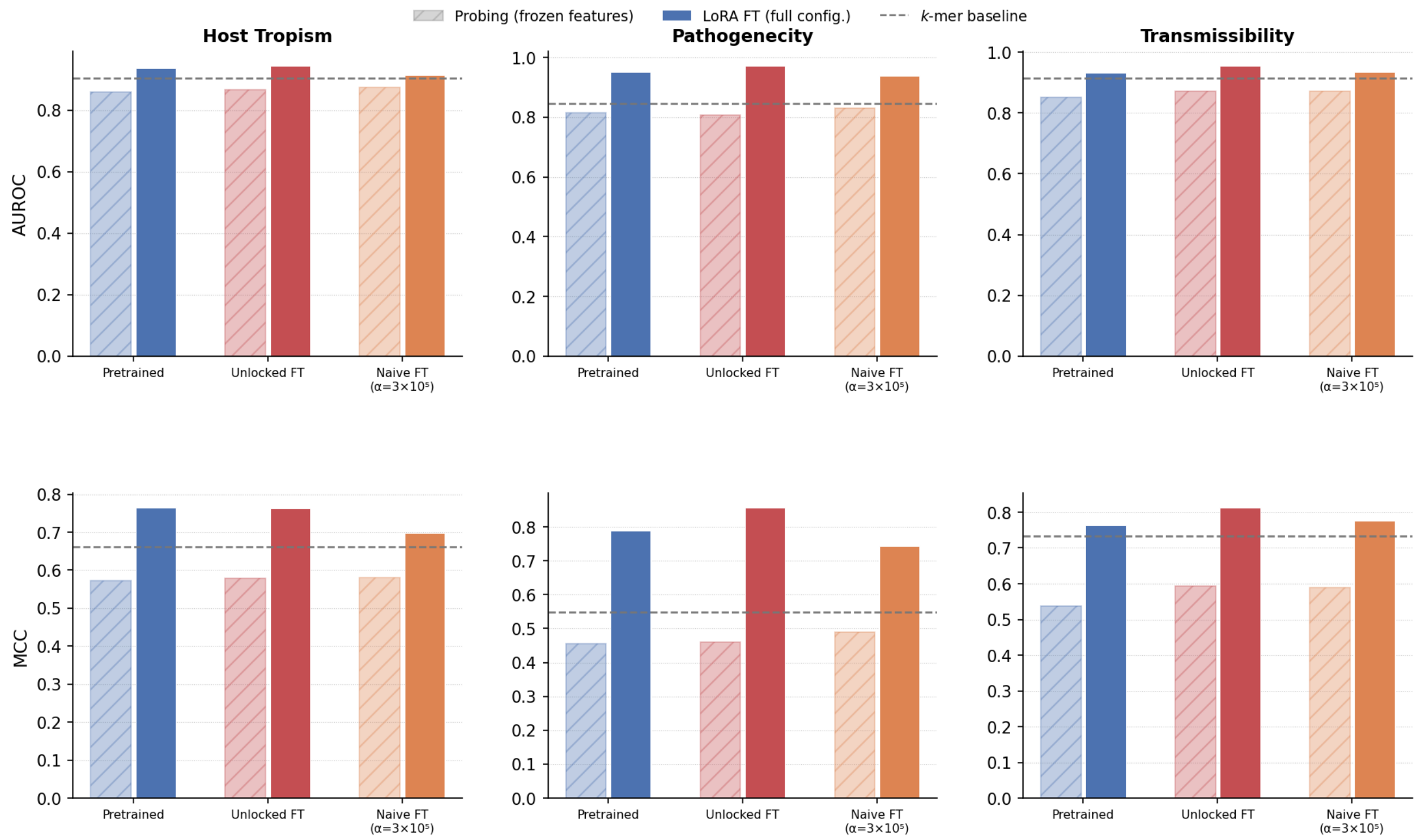

Figure S2: Probing is composition-confounded; fine-tuning is not. Mean HVUE AUROC **(a)** and MCC **(b)** for the pretrained, unlocked, and naively-attacked checkpoints under two evaluation protocols — frozen-feature linear probing (left bars) and LoRA fine-tuning (right bars) — with the 4-mer composition baseline as a dotted line (AUROC 0.903/0.846/0.914; MCC 0.662/0.549/0.733). Under probing, the k-mer baseline exceeds probed Evo on all tasks and the naively-attacked checkpoint scores above the unlocked reference, an impossible ordering. Under LoRA fine-tuning, every Evo checkpoint clears the k-mer baseline and the ordering is consistent with attack strength. Perplexity is not shown; this figure concerns the capability axis only.

bioRxiv preprint doi: https://doi.org/10.64898/2026.07.07.736795; this version posted July 10, 2026. The copyright holder for this preprint (which was not certified by peer review) is the author/funder, who has granted bioRxiv a license to display the preprint in perpetuity. It is made available under a CC-BY 4.0 International license.

Table S1: Held-out viral perplexity for all locking conditions. PPL values from the held-out evaluation set (366 genomes, standard windowing). Attacks A, D, E, G target $\alpha = 10^4$; B, C, C′, F, H, I, J target $\alpha = 3\times10^4$; K, L target $\alpha = 10^5$; M, N target $\alpha = 3\times10^5$. Attack C diverged (gradient norm = ∞ from step 100). All locking runs are single-seed. Downstream capability (AUROC/MCC) for these conditions under LoRA fine-tuning is reported in Table 1 and Fig. 1; probing-based AUROC values are omitted as composition-confounded (see Fig. S2).

**Condition**	α	**PPL**
Reference conditions		
Pretrained (no FT)	—	3.729
Locked, no FT	3×10^4	3.729
Unlocked FT	—	3.487
Naive fine-tuning (varied learning rate)		
A. Naive FT ($\eta = 10^{-6}$)	10^4	3.753
B. Naive FT ($\eta = 10^{-6}$)	3×10^4	3.816
C′. Naive FT ($\eta = 10^{-5}$)	3×10^4	3.776
C. Naive FT ($\eta = 10^{-4}$, aggressive)	3×10^4	diverged
Low-rank adaptation / bypass / SVD-chain		
D. LoRA r=16	10^4	3.729
E. B-bypass	10^4	4.091
F. B-bypass	3×10^4	4.123
H. SVD-chain k=2	3×10^4	3.745
I. SVD-chain k=3	3×10^4	3.705
J. SVD-chain k=5	3×10^4	3.760
Lock-strength robustness (α-scaling)		
K. Naive FT ($\eta = 10^{-5}$)	10^5	3.798
L. Naive FT ($\eta = 10^{-6}$)	10^5	3.876
M. Naive FT ($\eta = 10^{-5}$)	3×10^5	3.810
N. Naive FT ($\eta = 10^{-6}$)	3×10^5	5.861

Table S2: Computational and memory overhead per attack configuration. Parameter counts from model architecture summaries. Step times averaged over steps 100–500 on a single NVIDIA A100 80 GB, with 8-bit AdamW (Dettmers et al., 2022) and fp32 weights. Wall-clock multiplier is the per-step time relative to the unlocked fine-tuning baseline (3.27 s). Extra GPU memory is a conservative lower bound computed from extra parameters introduced by each attack: 10 B per extra trainable parameter above the unlocked baseline (4 B weight + 4 B gradient + 2 B 8-bit optimiser state) plus 4 B per extra non-trainable parameter (forward-pass storage). Activation memory, KV-cache, and framework overheads are not included. LoRA and bypass are listed as "—" for extra memory because the number of *trainable* parameters is far smaller than the unlocked baseline, so they cost *less* memory than legitimate fine-tuning despite adding total parameters.

**Configuration**	**Total (M)**	**Trainable (M)**	**Train.%**	**Extra (M)**	**Step (ms)**	**×baseline**	**Extra mem.**
Pretrained (unlocked baseline)	6 452.8	6 450.7	99.97%	0.0	3 267	1.00×	—
Locked — naive FT	6 989.7	6 987.6	99.97%	536.9	5 429	1.66×	+5.4 GB
LoRA (r=16) + locked	7 021.1	31.5	0.45%	568.3	3 950	1.21×	—
B-injection bypass	6 989.7	536.9	7.68%	536.9	2 243	0.69×	—
SVD-chain k=2	7 526.5	6 987.6	92.84%	1 073.7	5 236	1.60×	+8.1 GB
SVD-chain k=3	8 063.4	7 524.4	93.32%	1 610.6	6 238	1.91×	+12.9 GB
SVD-chain k=5	9 137.1	8 598.2	94.10%	2 684.4	8 215	2.51×	+23.6 GB
